# Transmissibility of hand, foot, and mouth disease in 97 counties of China

**DOI:** 10.1038/s41598-022-07982-y

**Published:** 2022-03-08

**Authors:** Wei Zhang, Jia Rui, Xiaoqing Cheng, Bin Deng, Hesong Zhang, Lijing Huang, Lexin Zhang, Simiao Zuo, Junru Li, XingCheng Huang, Yanhua Su, Benhua Zhao, Yan Niu, Hongwei Li, Jian-li Hu, Tianmu Chen

**Affiliations:** 1 State Key Laboratory of Molecular Vaccinology and Molecular Diagnostics, School of Public Health, Xiamen University, 4221-117 South Xiang’an Road, Xiang’an District, Xiamen City, Fujian Province People’s Republic of China; 2 Jiangsu Provincial Center for Disease Control and Prevention, 172, Jiangsu 21, Rd, Nanjing City, 210009 Jiangsu Province People’s Republic of China; 3 Chinese Center for Disease Control and Prevention, Beijing City, People’s Republic of China

**Keywords:** Diseases, Medical research, Risk factors

## Abstract

Hand, foot, and mouth disease (HFMD) is a serious disease burden in the Asia–Pacific region, including China. This study calculated the transmissibility of HFMD at county levels in Jiangsu Province, China, analyzed the differences of transmissibility and explored the possible influencing factors of its transmissibility. We built a mathematical model for seasonal characteristics of HFMD, estimated the effective reproduction number (*R*_*eff*_), and compared the incidence rate and transmissibility in different counties using non-parametric tests, rapid cluster analysis and rank-sum ratio in 97 counties in Jiangsu Province from 2015 to 2020. The average daily incidence rate was between 0 and 4 per 100,000 people in Jiangsu Province from 2015–2020. The Quartile of *R*_*eff*_ in Jiangsu Province from 2015 to 2020 was 1.54 (0.49, 2.50). Rugao District and Jianhu District had the highest transmissibility according to the rank-sum ratio. *R*_*eff*_ generally decreased in 2017 and increased in 2018 in most counties, and the median level of *R*_*eff*_ was the lowest in 2017 (*P* < 0.05). The transmissibility was different in 97 counties in Jiangsu Province. The reasons for the differences may be related to the climate, demographic characteristics, virus subtypes, vaccination, hygiene and other infectious diseases.

## Introduction

Hand, foot, and mouth disease (HFMD) is an infectious disease caused by enteroviruses^1^. The virus is mainly transmitted via the fecal–oral route, and can cause low-grade fever, maculopapular or papulovesicular rashes on the hands and soles of the feet, and painful oral ulcers^2^. The disease mainly occurs in children under the age of 10 years, especially in children aged 5–6 years^3^. HFMD has become widespread all over the world, Asia has a high incidence rate of HFMD^4^. HFMD was classified as a class C legal infectious disease with the highest incidence rate among all of the diseases that have been reported in China, infecting 2 million children each year^5^. Therefore, it is particularly important to study the incidence, transmission characteristics, and influencing factors of HFMD and to find appropriate prevention and control measures.

The transmission dynamics model can be used to study the transmissibility and influencing factors of HFMD^6–10^. Most of these studies focused on a large region such as a country or a province, and the results of these studies did not further explore the transmissibility and influencing factors in different counties in each country or province. To further explore the transmissibility and the influencing factors of HFMD in different counties, we used the incidence data of 2015–2020 HFMD cases in Jiangsu Province.

Jiangsu Province was chosen as our research area for the following reasons: First of all, at the time of writing, Jiangsu Province has become China's highest level of provincial comprehensive development and has entered the "middle and upper" developed countries level. The province has a high incidence (145.39/100,000)^11^ of HFMD, which result in a higher public health burden. . Secondly, Jiangsu province is currently divided into three regions (Central Jiangsu, Northern Jiangsu, and Southern Jiangsu). The three regions are bounded by the Huaihe River and irrigation canal, and the climate and social, and economic strength of the three regions have significant regional characteristics. These differences in climate, social and economic factors affect the spread of HFMD^12^.

We built a seasonal susceptible-exposed-infectious-asymptomatic-removed (SEIAR) model to fit the incidence rate of HFMD and then calculated the change in the HFMD transmissibility of 97 counties in Jiangsu from 2015 to 2020. Finally, we compared the transmissibility of HFMD between the three regions and between the 97 counties in those three regions in Jiangsu Province and analyzed the influencing factors of the transmissibility to provide a reference for controlling the outbreak of HFMD.

## Result

### County-level incidence map of HFMD in Jiangsu Province from 2015–2020

The average daily incidence of HFMD in various counties in Jiangsu Province ranged from 0 per 100,000 to 4 per 100,000. The median average daily incidence rate (0.5 per 100,000) was the highest in 2018. The median average daily incidence rate (0.003 per 100,000) was the lowest in 2020. In comparing the average daily incidence rate in Jiangsu Province in 2015–2020 with those in 2009–2013^21^ (excluding that in 2020, which smaller than in the previous years), the average daily incidence rate had a larger range, and the highest daily average incidence rate was 6.67 times the highest in 2009–2013.

**Figure 1:**
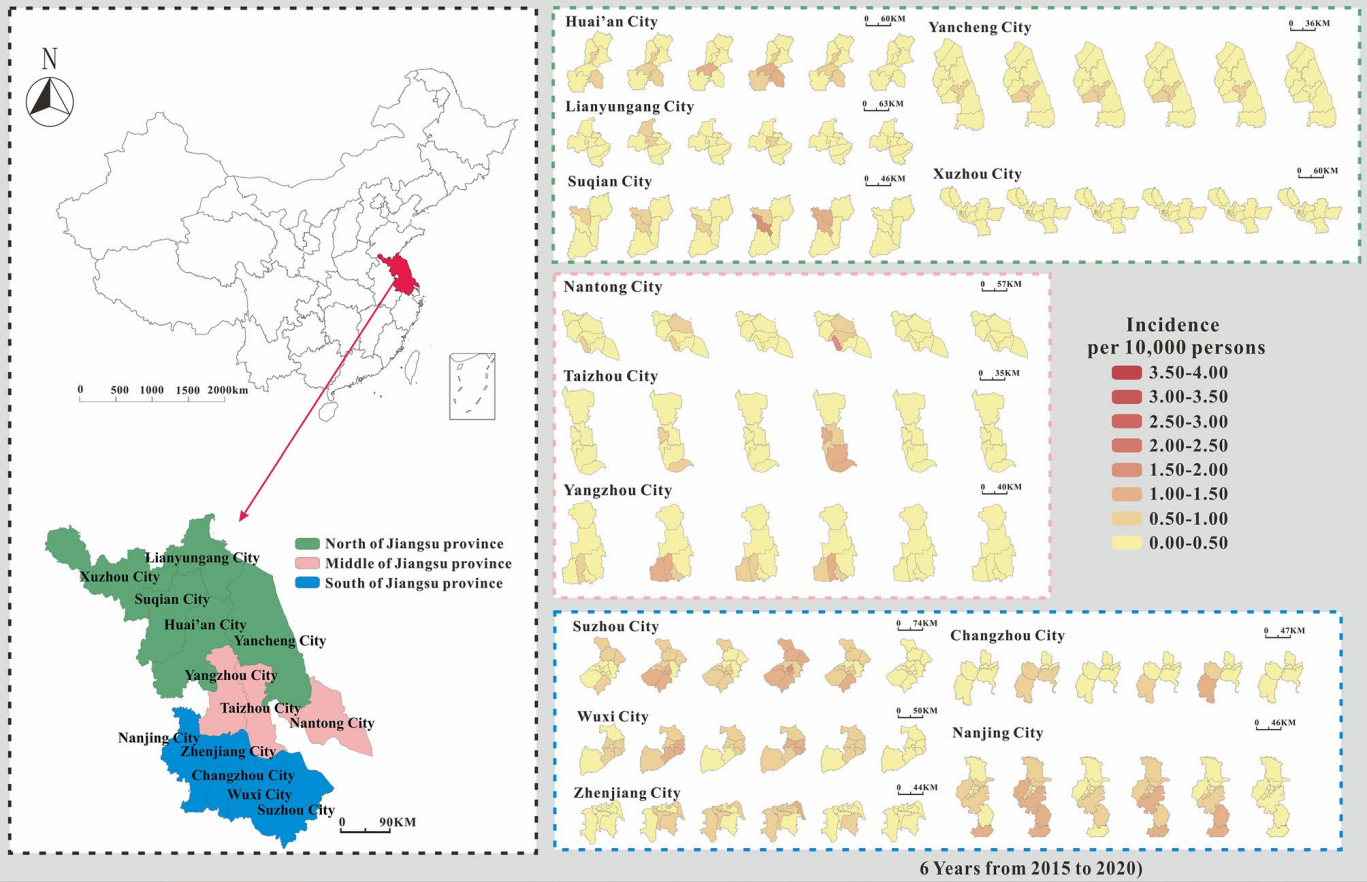
Map of average daily morbidity in Jiangsu Province form 2015–2020.

**Figure 2:**
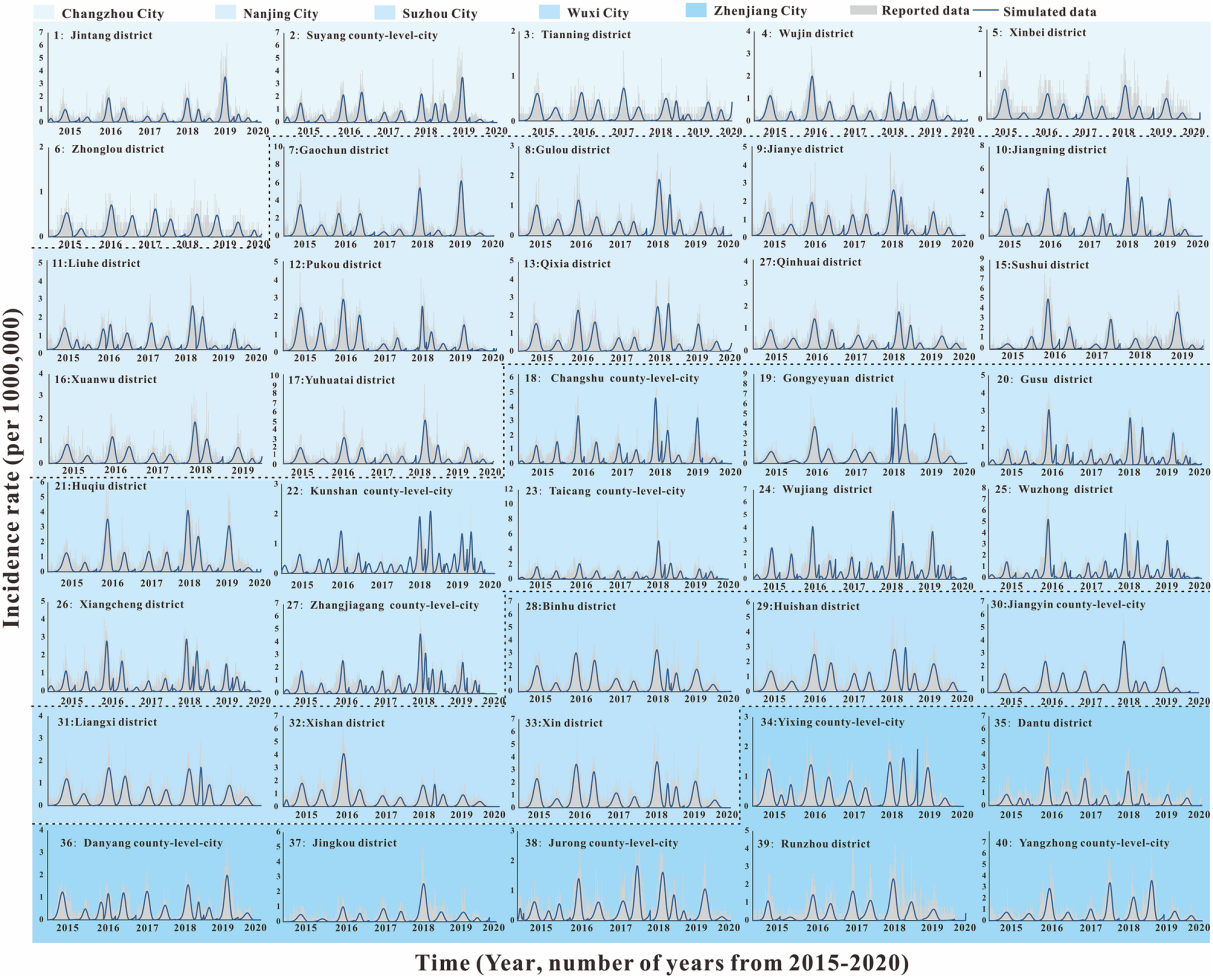
The simulated incidence rates of HFMD in different regions in Southern Jiangsu. No. 1–40 refers to Southern Jiangsu, including Changzhou city No. 1–6 (Jintang district, Suyang county-level-city, Tianning district, Wujin district, Xinbei district, Zhonglou district, respectively), Nanjing city No. 7–17 (Gaochun district, Gulou district, Jianye district, Jiangning district, Liuhe district, Pukou district, Qixia district, Qinhuai district, Sushui district, Xuanwu district, Yuhuatai district, respectively), Suzhou city No.18–27 (Changshu county-level-city, Gongyeyuan district, Gusu district, Huqiu district, Kunshan county-level-city, Taichng county-level-city, Wujiang district, Wuzhong district, Xiangcheng district, Zhangjiagang county-level-city, respectively), Wuxi city No. 28–34 (Binhu district, Huishan district, Jiangyin county-level-city, Liangxi district, Xishan district, Xin district, Yixing county-level-city, respectively) and Zhenjiang city No. 35–40 (Dantu district, Danyang county-level-city, Jingkou district, Jurong county-level-city, Runzhou district, Yangzhong county-level-city, respectively).

**Figure 3:**
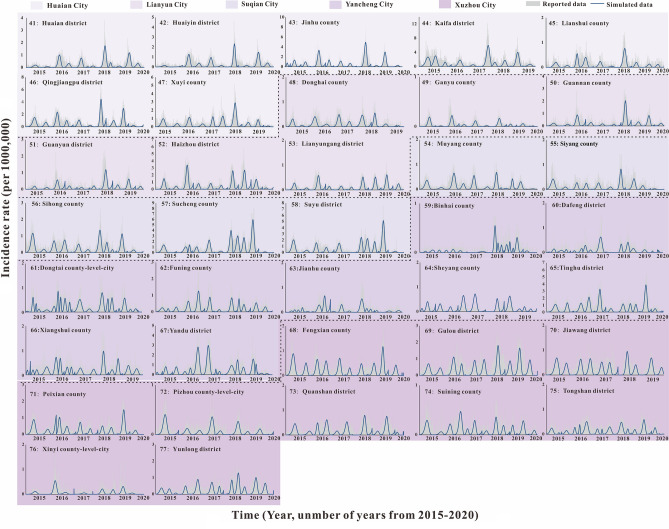
The simulated incidence rates of HFMD in different regions in Northern Jiangsu. No. 41–77 refers to Northern Jiangsu, including Huaian city No. 41–47 (Huaian district, Huaiyin district, Jinhu county, Kafaqu district, Lianshui county, Qingjiangpu district, Xuyi county, respectively), Lianyun city No. 48–53 (Donghai county, Ganyu county, Guannan county, Guanyun district, Haizhou district, Lianyungang district, respectively), Suqian city No. 54–58 (Muyang county, Siyang county, Sihong county, Sucheng district, Suyu district, respectively), Yancheng city No. 59–67 (Binhai county, Dafeng district, Dongtai county-level-city, Funing county, Jianhu county, Sheyang county, Tinghu district, Xiangshui county, Yandu district, respectively), Xuzhou city No. 68–77 (Fengxian county, Gulou district, Jiawang district, Peixian county, Pizhou county-level-city, Quanshan district, Suining county, Tongshan district, Xinyi county-level-city, Yunlong district, respectively).

**Figure 4:**
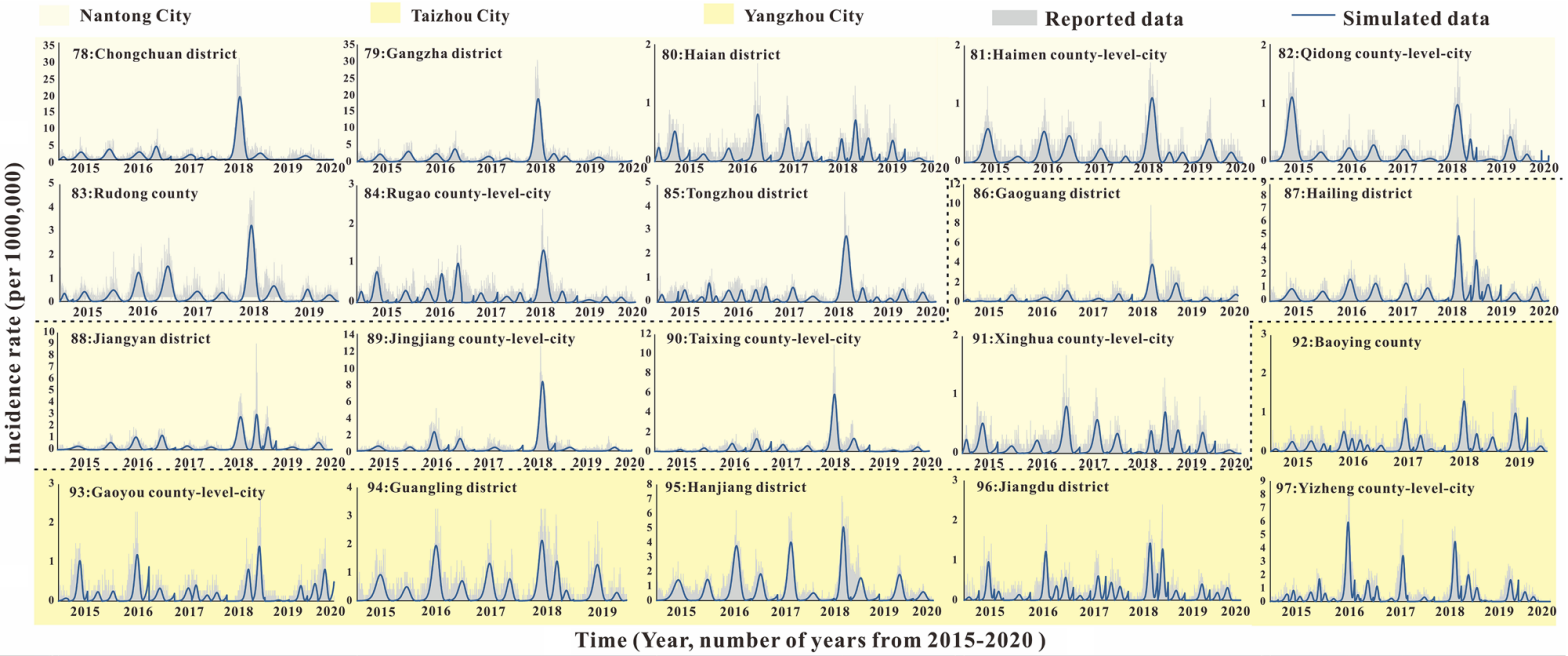
The simulated incidence rates of HFMD in different regions in Central Jiangsu. No. 78–97 refers to Central Jiangsu, including Nantong city No. 78–85 (Chongchun district, Gangzha district, Haian county-level-city, Haimen district, Qidong county-level-city, Rudong county, Rugao county-level-city, Tongzhou district, respectively), Taizhou city No. 86–91 (Gaogang district, Hailing district, Jiangyan district, Jingjiang county-level-city, Taixing county-level-city, Xinghua, county-level-city respectively), Yangzhou city No. 92–97 (Baoying county, Gaoyou county-level-city, Guangling district, Hanjiang district, Jiangdu district, Yizheng county-level-city, respectively).

According to the incidence map (Fig. 1), we found that in 2020, the average daily incidence rate of three regions (Southern Jiangsu, Northern Jiangsu and Central Jiangsu) was in the range of 0 per 100,000 to 0.5 per 100,000; from 2015 to 2019, the average daily incidence rate in Southern Jiangsu was generally more higher than that in Northern Jiangsu and Central Jiangsu. As shown in Figs. 2, 3, 4, we found that the HFMD outbreaks in Jiangsu Province showed obvious seasonality. The outbreaks in Southern Jiangsu occurred in two seasons per year (April to August and October to November), and the peak incidence rate and duration of the two outbreaks were relatively consistent. Similarly, the counties in Central Jiangsu experienced outbreaks during two seasons per year (April to August and October to November), the peak incidence rate and duration of the outbreak during these the two seasons per year were relatively consistent, but the peak height of the outbreak in 2018 was significantly higher than that in other years. Outbreaks in Northern Jiangsu were more complex. The counties in three major cities (Huai'an, Lianyungang, and Suqian) showed a trend of seasonal outbreaks (April to July). The counties in Yancheng City showed 2–3 outbreaks per year(March to May, June to July or September to November)and the counties in the Xuzhou City showed a steady two-season outbreak (April to August and October to November).

We found that most counties (84.54%, 82/97counties) had an average daily incidence rate that increased one year and decreased in the next, descending significantly in 2017 and increasing significantly in 2018. Based on the change in the average daily incidence rate in the region from 2015 to 2020, we divided the 97 counties into three typical situations via fast cluster analysis. The first type was characterized by an average daily incidence at a high level, maintained at 1 per 100,000. The incidence rate in 2018 was almost higher than that in 2016. The medium HFMD epidemic counties were the second type. The average incidence rate of HFMD in the middle epidemic counties was basically in the range of 0.5 per 100,000 to 1 per 100,000 in 2015–2019. The highest rates in 2016 and 2018 (approximately twice that of the years before and after). Low-incidence HFMD counties were the third type. The average HFMD incidence rate in 2015–2019 in the low epidemic counties was in the range of 0.01 per 100,000 to 0.5 per 100,000. Among them, the three counties with the lowest incidence rates were Binhai (0.01 per 100,000 to 0.10 per 100,000), Pizhou (0.06 per 100,000 to 0.11 per 100,000) and Xinqi (0.09 per 100,000 to 0.15 per 100,000). Almost all of the low-incidence counties showed the highest rates in 2016 and 2018 (maintained at 0.10 per 100,000). Counties seldomly showed that a downward trend after 2017, though this was found in some counties in Yancheng City. Very few counties (Gulou and Jiawang counties in Xuzhou City) showed an upward trend after 2017. (Fig. 1).

### Fitting results of SEIAR model of HFMD in Jiangsu Province from 2015 to 2020

The fitting results of the daily incidence rate over time in the 97 counties in Jiangsu Province from 2015 to 2020 are shown in Fig. 2, 3, 4. The correlation analysis between the fitting value and the actual reported value showed that the mean of the coefficient of correlation *R*^2^ was 0.50 ± 0.15, showing that the model was fitted well (Table S1).

### Transmissibility of HFMD in Jiangsu Province from 2015 to 2020

**Figure 5:**
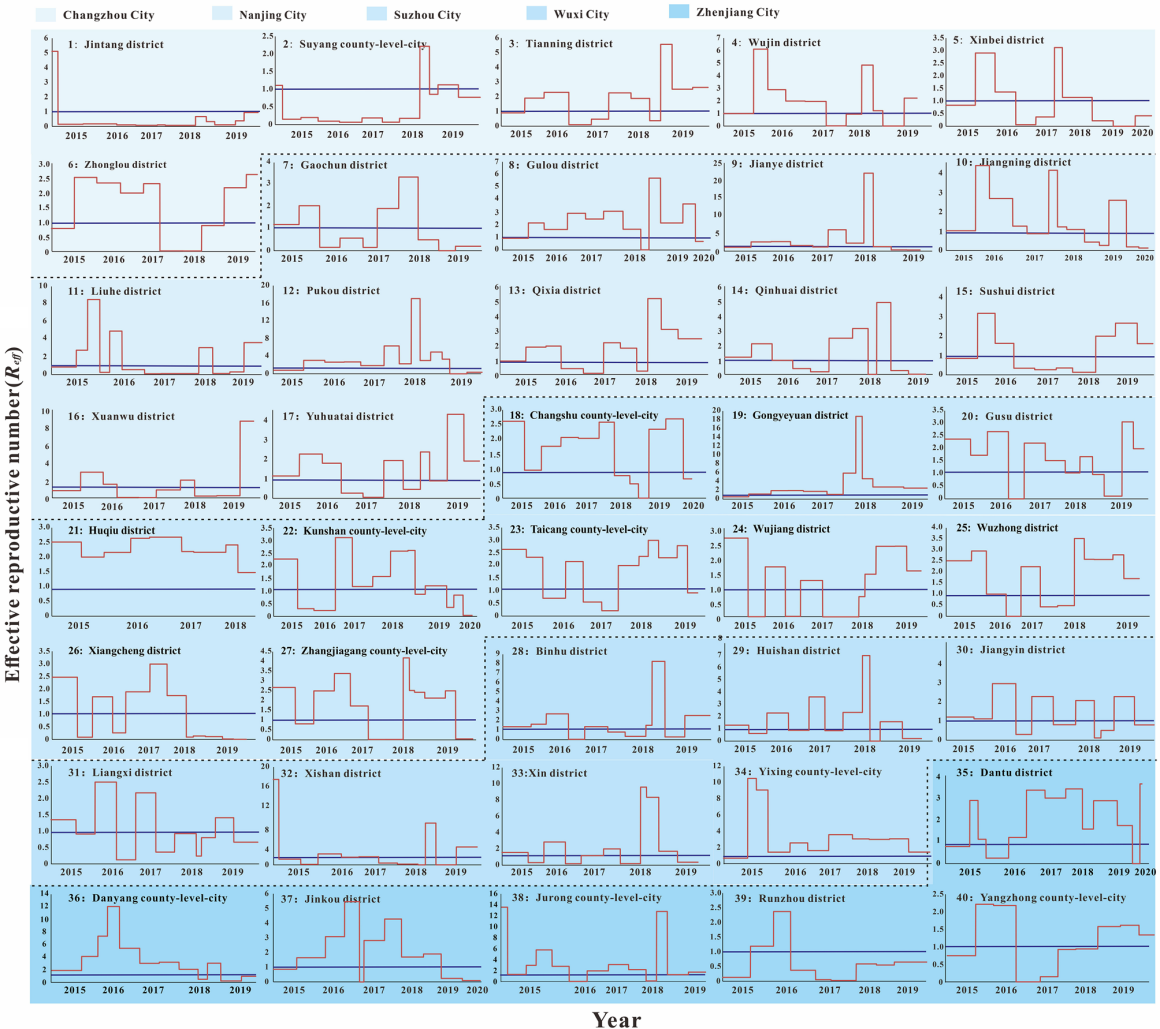
The effective reported number of HFMD in different regions in Southern Jiangsu. No. 1–40 refers to Southern Jiangsu, including Changzhou city No. 1–6 (Jintang district, Suyang county-level-city, Tianning district, Wujin district, Xinbei district, Zhonglou district, respectively), Nanjing city No. 7–17 (Gaochun district, Gulou district, Jianye district, Jiangning district, Liuhe district, Pukou district, Qixia district, Qinhuai district, Sushui district, Xuanwu district, Yuhuatai district, respectively), Suzhou city No.18–27 (Changshu county-level-city, Gongyeyuan district, Gusu district, Huqiu district, Kunshan county-level-city, Taichng county-level-city, Wujiang district, Wuzhong district, Xiangcheng district, Zhangjiagang county-level-city, respectively), Wuxi city No. 28–34 (Binhu district, Huishan district, Jiangyin county-level-city, Liangxi district, Xishan district, Xin district, Yixing county-level-city, respectively) and Zhenjiang city No. 35–40 (Dantu district, Danyang county-level-city, Jingkou district, Jurong county-level-city, Runzhou district, Yangzhong county-level-city, respectively).

**Figure 6:**
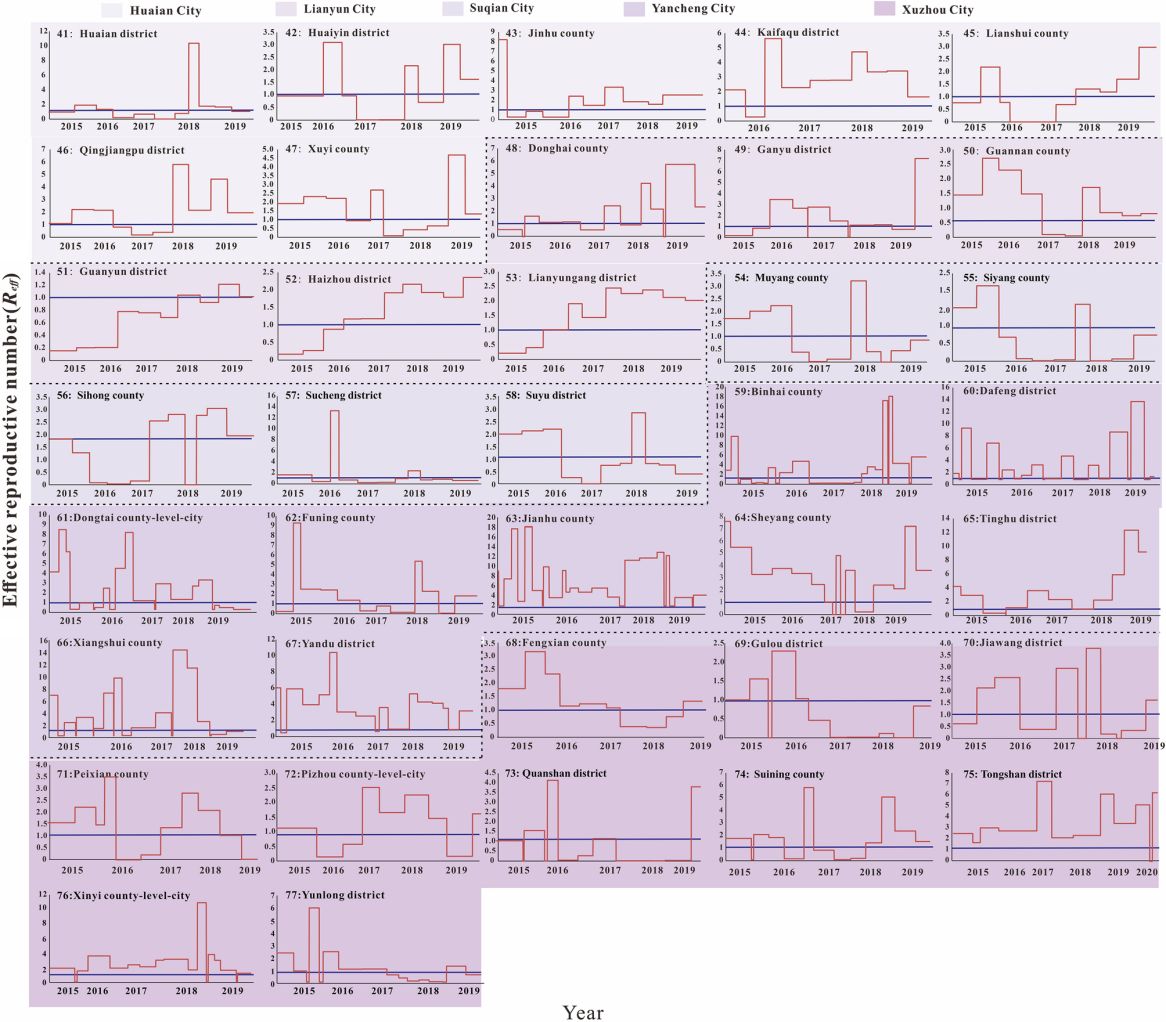
The effective reported number of HFMD in different regions in Northern Jiangsu. No. 41–77 refers to Northern Jiangsu, including Huaian city No. 41–47 (Huaian district, Huaiyin district, Jinhu county, Kafaqu district, Lianshui county, Qingjiangpu district, Xuyi county, respectively), Lianyun city No. 48–53 (Donghai county, Ganyu county, Guannan county, Guanyun district, Haizhou district, Lianyungang district, respectively), Suqian city No. 54–58 (Muyang county, Siyang county, Sihong county, Sucheng district, Suyu district, respectively), Yancheng city No. 59–67 (Binhai county, Dafeng district, Dongtai county-level-city, Funing county, Jianhu county, Sheyang county, Tinghu district, Xiangshui county, Yandu district, respectively), Xuzhou city No. 68–77 (Fengxian county, Gulou district, Jiawang district, Peixian county, Pizhou county-level-city, Quanshan district, Suining county, Tongshan district, Xinyi county-level-city, Yunlong district, respectively).

The Quartile of *R*_*eff*_ in Jiangsu Province from 2015 to 2020 was 1.54 (0.49, 2.50), the 95% reference range was less than 5.88, and the highest *R*_*eff*_ could reach 20,000 times the lowest. *R*_*eff*_ showed a periodic change in the unit of year, and there was at least one *R*_*eff*_ peak in the adjacent years, with the peak greater than 1.0. The median of *R*_eff_ for each year from 2015 to 2019 was different (*χ*^2^ = 21.283, *P* = 0.000), and that the median of *R*_*eff*_ in 2017 was smaller than that in other years (*P* < 0.05). The median of *R*_*eff*_ in Southern Jiangsu was the smallest among the three regions (*P* < 0.05), and that the median *R*_*eff*_ of Changzhou City was lowest compared with other four cities in Southern Jiangsu (*P* < 0.05) (Fig. 5). The median of *R*_*eff*_ of Yancheng City was the highest compared with other four cities in Northern Jiangsu (*P* < 0.05) (Fig. 6).

**Figure 7:**
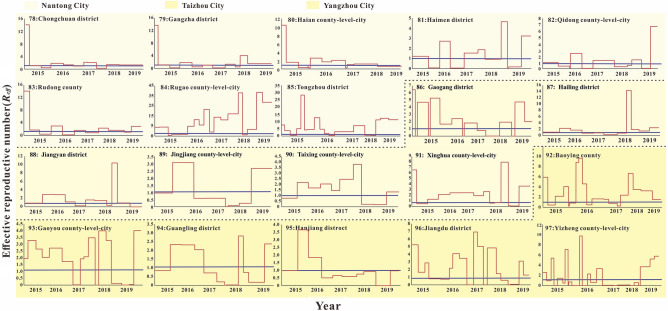
The effective reported number of HFMD in different regions Central Jiangsu. No. 78–97 refers to Central Jiangsu, including Nantong city No. 78–85 (Chongchun district, Gangzha district, Haian county-level-city, Haimen district, Qidong county-level-city, Rudong county, Rugao county-level-city, Tongzhou district, respectively), Taizhou city No. 86–91 (Gaogang district, Hailing district, Jiangyan district, Jingjiang county-level-city, Taixing county-level-city, Xinghua, county-level-city respectively), Yangzhou city No. 92–97 (Baoying county, Gaoyou county-level-city, Guangling district, Hanjiang district, Jiangdu district, Yizheng county-level-city, respectively).

According to the periodic change of *R*_*eff*_ in different counties, 97 counties could be divided into five types as the following: (1) *R*_*eff*_ was basically at a high level and remained above 1.0 with a cyclical change, represented by Huqiu (No.21), Dafeng (No.60), Rugao (No.84), and so on; (2) *R*_*eff*_ increased significantly in 2018 with the highest peak height, represented by Suyang (No.2), Huaian(No.41), Hailing(No.87) , and so on; (3) *R*_*eff*_ in 2015–2016 were basically at a high level, but the *R*_*eff*_ values in 2017–2019 had a downward trend, represented by Liuhe (No.11), Liangxi (No.31), and Donghai(No.61), and so on; (4) *R*_*eff*_ was approximately 1.0 after 2015, represented by Yixing (No.34), Fengxian (No.68), Chongchun (No.78), and so on; (5) *R*_*eff*_ changed periodically at an estimated 1.0 level from 2016 to 2018, but rose abruptly in 2019, represented by Taining (No.3), Xuanwu (No.16), Donghai(No.82) , and so on (Figs. 5, 6, 7).

### Comparison of the transmissibility in the 97 counties

We compared the transmissibility of 97 counties using *RSR*. According to the *RSR* distribution table (Table 1), we constructed the *RSR* and probit regression equations which could be obtained as $\widehat{{\text RSR}} = - 0.261 + 0.151 \times {{Probit}}$ (*F* = 1813.37, *P* = 0.000), and the $\widehat{{{\text RSR}}}$ of each district was calculated and used to classify the transmissibility into six levels, as showed in Table 2. Transmissibility became increasingly weaker from 1 to 6. The result showed that counties with the strongest transmissibility were Rugao in Central Jiangsu and Jianhu in Northern Jiangsu, while the weakest were Liyang and Jintan in Southern Jiangsu and Sihong in Northern Jiangsu. Most of the counties were in levels 3–4, indicating that the transmissibility of these counties was relatively similar, especially in the same region or city.

**Table 1:** The distribution of *RSR.*

*RSR*	*f*	*∑f*	*R*	*R`*	(*R`*/n)*100%	Probit
0.1	1	1	1	1	1.031	2.685
0.11	1	2	2	2	2.062	2.959
0.16	1	3	3	3	3.093	3.133
0.18	1	4	4	4	4.124	3.263
0.23	1	5	5	5	5.155	3.370
0.26	1	6	6	6	6.186	3.461
0.27	1	7	7	7	7.216	3.540
0.28	1	8	8	8	8.247	3.611
0.29	1	9	9	9	9.278	3.676
0.3	1	10	10	10	10.309	3.736
0.31	2	12	11, 12	11.5	11.856	3.818
0.33	1	13	13	13	13.402	3.892
0.35	1	14	14	14	14.433	3.939
0.37	1	15	15	15	15.464	3.983
0.38	3	18	16, 17, 18	17	17.526	4.066
0.39	3	21	19, 20, 21	20	20.619	4.180
0.4	2	23	22, 23	22.5	23.196	4.268
0.42	2	25	24, 25	24.5	25.258	4.334
0.43	2	27	26, 27	26.5	27.320	4.397
0.45	2	29	28, 29	27.5	28.351	4.428
0.46	1	30	30	30	30.928	4.502
0.47	4	34	31, 32, 33, 34	32.5	33.505	4.574
0.48	3	37	35, 36, 37	36	37.113	4.671
0.49	2	39	38, 39	38.5	39.691	4.739
0.5	2	41	40, 41	40.5	41.753	4.792
0.51	5	46	42, 43, 44, 45, 46	44	45.361	4.883
0.52	3	49	47, 48, 49	48	49.485	4.987
0.53	5	54	50, 51, 52, 53, 54	52	53.608	5.091
0.54	4	58	55, 56, 57, 58	56.5	58.247	5.208
0.55	8	66	59, 60, 61, 62, 63, 64, 65, 66	62.5	64.433	5.370
0.57	1	67	67	67	69.072	5.498
0.58	2	69	68, 69	68.5	70.619	5.542
0.59	2	71	70, 71	70.5	72.680	5.603
0.6	1	72	72	72	74.227	5.650
0.61	4	76	73, 74, 75, 76	74.5	76.804	5.732
0.62	1	77	77	77	79.381	5.820
0.64	1	78	78	78	80.412	5.856
0.65	7	85	79, 80, 81, 82, 83, 84, 85	82	84.536	6.017
0.66	3	88	86, 87, 88	87	89.691	6.264
0.67	1	89	89	89	91.753	6.389
0.68	2	91	90, 91	90.5	93.300	6.498
0.73	1	92	92	92	94.845	6.630
0.75	1	93	93	93	95.876	6.737
0.76	1	94	94	94	96.907	6.867
0.77	1	95	95	95	97.938	7.041
0.79	1	96	96	96	98.970	7.315
0.84	1	97	97	97	99.999	9.265

**Table 2:** Ranking of *R*_*eff*_ in 97 counties of Jiangsu Province from 2015 to 2020.

Grade	*P*_x_	Probit	$\widehat{\text RSR}$	District
1	< *P*_2.275_	< 3	< 0.192	Northern Jiangsu: Jianhu Central Jiangsu: Rugao
2	*P*_2.275_ ~	3 ~	0.192 ~	Southern Jiangsu: Pukou, Gulou Northern Jiangsu: Yandu, Tongshan, Tinghu, Sheyang, Xinyi, Xiangshui, Dafeng Central Jiangsu: Tongzhou, Baoying, Gaoyou, Jiangdu
3	*P*_15.866_ ~	4 ~	0.343 ~	Southern Jiangsu: Danyang,Yixing, Jiangning, Huqiu, Jurong, Liuhe, Xishan, Dantu, Kaifaqu, Gongyeyuan, Jingkou, Wuzhong, Gusu, Wujin, Zhangjiagong, Huishan, Taicang, Binhu, Jianye, Xin, Northern Jiangsu: Binhai, Jianhu, Ganyu, Qingjiangou, Dontai, Peixian, Funing, Suining, Dongahi Central Jiangsu: Yizheng, Gaogang, Xinghua, Rudong, Jiangyan
4	*P*_50_ ~	5 ~	0.494 ~	Southern Jiangsu: Qixia, Changshu, Yuhuatai, Tianning, Zhonglou, Kunshan, Jiangyin, Qinhuai, Xiangcheng, Liangxi Northern Jiangsu: Siyang, Xuyi, Lianyugang, Fengxian, Jiawang, Yunlong, Haizhou, Guannan, Huaiyin, pizhou,Suyu Central Jiangsu: Hailing, Chongchuan, Taixing, Haimen, Jingjiang, Guangling, Haian
5	*P*_84.134_ ~	6 ~	0.645 ~	Southern Jiangsu: Yangzhong, Sushui, Xuanwu, Wujiang, Gaocun, Xinbei, Gulou, Runzhou Northern Jiangsu: Huaian, Muyang, Quanshan, Sucheng, Lianshui, Guanyun Central Jiangsu: Qidong, Hanjiang
6	*P*_97.725_ ~	7 ~	> 0.796	Southern Jiangsu: Suyang, Jintang Northern Jiangsu: Sihong

### Sensitivity analysis

**Figure 8:**
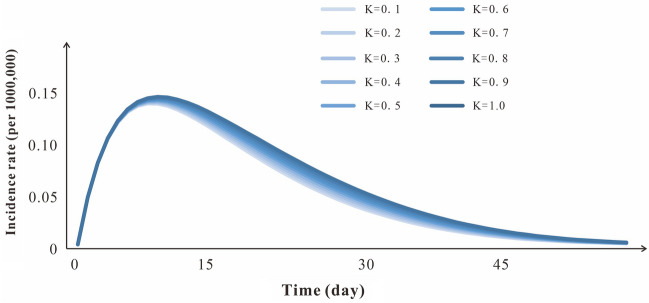
Sensitivity analysis of k (range 0–1).

We selected a period of incidence data to fit curve with 10 values of parameter *κ* in the range of 0–1. The result showed that the fitting values had a high degree of coincidence, which indicated that the SEIAR model was not sensitive to this study (Fig. 8).

## Discussion

In this study, the seasonally adjusted SRIAR model was used to study the transmissibility of HFMD among the 97 counties in Jiangsu Province, to provide suggestions for local Centers for Disease Control and Prevention, community in Jiangsu Province and other areas with a similar transmissibility of HFMD.

### Analysis of the different incidence rates and transmissibility in three regions

The incidence rate of Southern Jiangsu was higher than Northern Jiangsu and peaked in two seasons per year, which was consistent with earlier studies of HFMD in Jiangsu Province^21^. In this study, we found that some counties of Northern Jiangsu had one seasonal peak, two seasonal peak or more than two seasonal peaks. The average *R*_*eff*_ of HFMD in Jiangsu Province from 2015 to 2020 was 1.54, which was similar to the research results of studies on foreign and most domestic provinces and regions; while the *R*_*eff*_ was lower than that of Shenzhen, Guangdong Province^18^. We found that the *R*_*eff*_ in Southern Jiangsu was less than that in Northern Jiangsu, which was contrary to the incidence rate of the regions. We considered that the reasons for the different incidence rates, seasons and transmissibility in three regions were as following: (1) The climate zones of the regions were inconsistent may be influence the spread of HFDM virus and the epidemic virus serotypes. The different pathogens would compete with each other, resulting in the change in seasonal peaks and transmissibility. Liu et al. found that the incidence rate of HFMD in Jiangsu was proportional to the average temperature and rainfall but was negatively correlated with the days of rainfall (≥ 0.1 mm), low temperature, high temperature, and sunshine duration^21^. Moderately warm environment was found to promote the spread of the HFMD virus^22^. Our team previously used mathematical models to analyze the interaction of the main pathogens of Changsha HFMD and found that different pathogens would compete with each other, resulting in changing transmissibility and an impact on morbidity^23^. In recent years, studies found different disease prototypes in different urban areas of Jiangsu Province and determined that different dominant strains appear alternately^24,25^. We considered that Southern Jiangsu was warmer than Northern Jiangsu, which may have caused the incidence rate to be higher, and the winter in the northern region was too cold to prevent the spread of HFMD. The difference viruses between Northern Jiangsu and Southern Jiangsu, may be affected by the growth environment, and the transmissibility be affected by the interaction between viruses. (2) The demographic characteristics and the hygiene levels of the regions were different. The south of Jiangsu Province was a densely populated area, which had higher birth rates. Studies found that most of the infected patients in this area were infants and children under five years of age. While the medical and health development in Jiangsu Province was unbalanced^26–28^. The highest development of medical and health undertakings was located in Southern Jiangsu (Nanjing, Suzhou, and Wuxi), and the lowest health environment was mainly in Northern Jiangsu (Lianyun, Yancheng, and Suqian). Although the population base was larger in Southern Jiangsu which could have enabled greater opportunities for population contact and communication, economically developed areas had higher medical and health levels, higher health awareness, and a stronger ability to block the transmission of HFMD, making the transmissibility of Southern Jiangsu relatively weaker than that of Northern Jiangsu. (3) There was a small range of outbreaks. In Northern Jiangsu, for cities with low incidence rates, such as Yancheng (Binhai), the small number of incidences induced data instability in the transmission dynamics model analysis. The uncertainty of the results increased, coupled with the region with a long-term low incidence rate, poor health level, and insufficient experience in HFMD outbreaks, resulting in a strong transmissibility during the outbreak. Therefore, we thought that there may have been small ranges of outbreaks in Northern Jiangsu, some disadvantages of the kinetic model itself, and a weaker ability to cope with the outbreak, making the *R*_*eff*_ in the region higher. (4) Emerging contaminants influenced the antibody levels of HFMD virus in infants. PFASs can enter humans through biological enrichment of food (chain) webs in freshwater and marine environments^29^. A study showed that cord serum long-chain PFASs concentrations significantly correlated with low antibody levels of CA16 and EV71 at three months^30^. A high concentration of PFOS was found in the Yangtze Estuary sediments (72.9–536.7 ng/g)^31^. Southern Jiangsu is near the Yangtze Estuary, the immunity of infants with HFMD may be reduced by fluoride pollutants, leading to a higher probability of incidence.

Based on the aforementioned analysis, we suggested that Southern Jiangsu should pay more attention to a wide range of public health publicity during the seasons of HFMD onset. For the central and Northern Jiangsu areas with strong transmissibility of HFMD, improving health services, strengthening investments in healthcare and implementing protective measures that are more helpful in reducing its prevalence are necessary. A study showed that enteroviruses were detected on the surface of environmental items in hospitals and community public playgrounds in areas with a high incidence of HFMD in Jiangsu Province^32^, thus strengthening the need for disinfection during high-incidence periods. Communities are areas prone to cross-infection and should be taken seriously.

### Analysis of the different incidence rates and transmissibility in various years

The trend of HFMD transmissibility over time showed that *R*_*eff*_ was the lowest in 2017, which may have been related to the implementation of the EV71 vaccine in 2016^33,34^. Because we found that the incidence rate was also the lowest in 2017, the immunity provided by the vaccine against EV71 and the publicity of vaccination reduced the number of susceptible and infected people, thereby reducing the actual transmissibility of HFMD. It is interesting that in many counties, the incidence rates and transmissibility suddenly increased in 2018, and the peak height could have been higher than that in 2015 and 2016. We analyzed the different incidence rates and transmissibility in various years based on following aspects: (1) From the perspective of climate change, although the incidence and spread of HFMD are related to climate factors^21^, according to some meteorological studies, the temperature and rainfall in 2017 and 2018 were not abnormal compared with those in other years^35,36^. Therefore, compared to 2017, the average incidence rate in 2018 increased significantly, which may not have been related to climatic factors. (2) In terms of changes in epidemic virus subtypes or the change in transmissibility caused by a variety of viruses. The serotypes of HFMD viruses are extensive. Studies have shown that human enterovirus (HEV)-A includes CA2–8, 10, 12, 14, 16, and EV71^37,38^. Although EV71 and CA16 are the main causes of HFMD outbreaks, other HEV–A pathogens were found in sporadic HFMD cases^38,39^, In addition, some studies have reported that the basic reproduction number of different types of enterovirus is different, and the basic reproduction number of coxsackievirus is the highest^40^. Relevant studies have shown that CA6 has gradually become the main pathogen of HFMD in the world. The prevalence of Finland^41^, Spain^42^, the United States^43^ in Europe and Japan in Asia^44^increased to 70% or more from 2008 to 2011, and those in Guangdong^45^ and Changchun^45^ in China increased to more than 60% in 2013. According to the data from 2008 to 2010, EV71 and CA16 were the leading epidemics of HFMD in this province, accounting for nearly 1:11^12^. Recent studies also showed that EV71 and CA16 were the main pathogens of HFMD in Suzhou in 2017, and CA6 was the main pathogen of HFMD in 2018^46^, and the co-infection of EV71 or Cox A16 and CA6 or CA10 was also found in Suzhou^29^. Our team previously used mathematical models to analyze the interaction of the main pathogens of HFMD in Changsha City, and found that different pathogens would compete with each other, resulting in the change in transmissibility, EV71 interacts with CA16, and the interactions between EV71 and other enteroviruses and between CA16 and other enteroviruses are all directional^23^. Therefore, although EV71 vaccination began in 2016^33,34^, the current vaccine does not have any protective effect on CA16, CA6 and other subtypes^34^, the repeated outbreaks after 2017 may have been be caused by CA16, CA6 infection or new virus subtypes after vaccination, or the result of the different pathogens compete with each other. (3) Regarding the impact of other infectious diseases, in this study, we found that the average daily incidence rate of HFMD in the first half of 2020 was 10 times lower than that before. This indicated that the protective measures against coronavirus disease (COVID-19), such as school closures, business discontinuation, frequent hand washing and wearing of masks, and maintaining social distance, have affected the prevalence of HFMD to some extent. Other research also showed that the incidence rate of HFMD was affected by road passenger volume and population mobility during the school terms and Spring Festival. The combined effect was more significant than that of meteorological factors on the epidemic of HFMD^47^. (4) From the perspective of air pollution, studies found that air pollution (CO, SO_2_, NO_2_, O_3_, and PM2.5) has a short-term impact effect on the incidence rate of HFMD^48–51^. While there was no clear conclusion on the impact of different pollutants on HFMD. According to the results of the monthly and interannual variation of persistent air pollution events from December 2, 2013 to December 31, 2018 and the spatiotemporal distribution characteristics of air quality index, Jiangsu Province had the worst air pollution in winter and lowest pollution in summer; the annual ρ (PM2.5), (PM10), (SO_2_), and (CO) decreased year by year, and the maximum 8 h of ρ (O_3_) increased year by year, with no significant annual change in ρ (NO_2_)^52^. But the peak incidence rates of HFMD every year in Jiangsu province was in spring, summer, and autumn, with better air quality. We thought that the impact of air quality on HFMD in different years in Jiangsu is not clear.

Therefore, we suggest that based on the classification of the different transmissibility described by results, specific counties should be selected to monitor the subtypes of HFMD, and an HFMD vaccine for different subtypes should be developed to cope with the changes in epidemic pathogens.

### Analysis of the comprehensive comparison results of transmissibility in the 97 counties

Jianhu District in Northern Jiangsu and Rugao District in Central Jiangsu had the strongest comprehensive evaluation of transmissibility, though the trends of transmissibility were different. Jianhu District maintained a high transmissibility from 2015 to 2016 and had a downward trend from 2017 to 2019. Based on previous research, we found that in Yancheng City, where Jianhu is located, HFMD was highly prevalent among infants and that the higher the level of maternal antibodies against to EV71, the stronger the protection for infants^53^. Therefore, the implementation of vaccine immunization in Jianhu District provided a certain protective effect. However, it is still necessary to further strengthen the propaganda and education and detect whether there is a new virus subtype epidemic. The transmissibility in Rugao District had an uptrend trend from 2017 to 2020, with research showing that the HFMD in Rugao District had been more serious in recent years, and the incidence rate and incidence ratio were the highest fpr class C infectious diseases^54^, with critically ill infected patients in 2015 to 2020. There were inappropriate nursing practices and poor health environments in the rural areas in this district, and the number of vaccination individuals was low. We need to focus on improving the health environment, strengthening public health marketing and healthcare education, improving the awareness of epidemic prevention, and improving epidemic situation monitoring, especially the analysis and monitoring of virus subtypes of severe patients.

### Limitations

Owing to shortcomings in the data, this study had some limitations. In this model, factors that may affect the transmissibility, such as age and gender, were not included, which may have impacted the results. The actual data of possible influencing factors, such as climate characteristics, virus types, population data, were also not collected for a correlation analysis with transmissibility of HFMD in various counties. We did not consider that the infected individuals were also affected by other bacterial or viruses, which affected the course of HFMD. Additionally, we did not consider that these infectious individuals could be reinfected with HFMD after recovering from HFMD.

## Conclusion

1. The epidemic situation of HFMD in Jiangsu Province in 2015–2019 was more severe than in 2009–2013. The impact of COVID-19 was related to a reduction in the HFMD incidence rate in Jiangsu Province in 2020.
2. The outbreaks and transmissibility of HFMD in Jiangsu had regional and seasonal characteristics. The higher the incidence rate in the three regions, the lower the transmissibility. The peak period of the epidemic changed from season to season.
3. The differences in the incidence rates and transmissibility of HFMD in Jiangsu Province were related to the climate, population, virus subtypes, vaccination, and other infectious diseases; the difference in virus subtypes may have been the most important factor.
4. Rugao District in Central Jiangsu and Jianhu District in Northern Jiangsu had the strongest transmissibility of HFMD among these 97 counties in Jiangsu Province. The vaccination rate should be increased in Jianhu District, and health marketing, health conditions, and virus subtype monitoring should be reinforced in Rugao District.
5. The transmissibility was similar between some cities or regions, suggesting that the representative areas should be selected for virus subtype surveillance according to the characteristics of transmissibility in Jiangsu Province.

## Methods

### Ethics declarations

This efort of disease control was part of CDC’s routine responsibility in Jiangsu Province, China. Therefore, institutional review and informed consent were not required for this study. All data analyzed were anonymized, and does not contain any personal privacy or identity information, so the ethics approval documents may be exempted.

### Data sources

Jiangsu Province is located in the eastern coastal area of the Chinese mainland. From Southern Jiangsu to Northern Jiangsu, the climate transition from subtropical zone to warm temperate zone, with mild climate and moderate rainfall. HFMD is a class C legal infectious disease in China. Doctors must report these cases with suspected HFMD, including suspected cases, clinical cases, and experimental diagnostic cases within 24 h to the network direct reporting system for the monitoring information of statutory infectious diseases. The data of the HFMD cases used in this study were obtained from the China Information System for Disease Control and Prevention, including the number of cases, deaths reported daily and date of onset. The case types included clinical diagnosis and laboratory diagnosis cases. Demographic information was obtained from the statistical yearbook of Jiangsu Province, including the number of permanent residents at the end of the year, birth rate, and mortality rate.

According to the statistical yearbook of Jiangsu Province, Jiangsu Province is divided into three regions. The three regions are bounded by the Huaihe River and irrigation canal, with a subtropical humid monsoon climate in the south and a warm temperate humid and semi-humid monsoon climate in the north. The size of three regions is similar, but there are obvious differences in social and economic development: the regional economy is the highest in Southern Jiangsu, followed by Central Jiangsu and then Northern Jiangsu^13^.

About 91.51% (97/106) counties in Jiangsu province was including, and these counties include counties, districts and county-level cities. Northern Jiangsu region includes Huai'an (7 counties), Lianyungang (6 counties), Suqian (5 counties), Yancheng (9 counties), and Xuzhou (10 counties), with a total of 37 counties; Central Jiangsu includes Nantong (8 counties), Taizhou (6 counties), and Yangzhou (6 counties), with a total of 20 counties; Southern Jiangsu includes Changzhou (6 counties), Nanjing (11 counties), Suzhou (10 counties), Wuxi (7 counties), and Zhenjiang (6 counties), with a total of 40 counties.

### Case definition

The diagnosis of HFMD was carried out according to the guideline issued by the National Health and Family Planning Commission of the People's Republic of China^14^. Patients with HFMD, whether probable or confirmed, were classified as severe if they had any neurological complications (aseptic meningitis, encephalitis, encephalomyelitis, acute flaccid paralysis, and autonomic nervous system dysregulation), or cardiopulmonary complications (pulmonary edema, pulmonary haemorrhage, and cardiorespiratory failure), or both; otherwise, patients were categorized as mild cases.

According to the diagnostic criteria (2018 version) of HFMD^14^, the confirmed cases were confirmed by via enzyme-linked immunosorbent assays (ELISA), reverse transcription polymerase chain reaction (RT-PCR), real-time PCR, or virus isolation.

### The transmission models of HFMD

According to the epidemiological feature of HFMD and our previous studies^6,7,9^, the SEIAR model could be used for the simulation in the model, the population was divided into susceptible individuals (S), exposed individuals (*E*), infectious individuals (*I*), asymptomatic individuals (*A*) and recovery individuals (*R*). The model diagram is shown in Fig. S1.

The differential equations of the model were used to describe the dynamic changes of each state. The corresponding model equations were as follows:

1. The model assumed that HFMD could not propagate vertically, and that all of the infectious individuals were infected through contact. Then we set birth rate (*br*), the natural mortality rate (*dr*), and the mortality rate of the infectious individuals (*f)*. The mortality rate of all kinds of people in the disease spectrum was low, and the mortality rate of population attributable to HFMD was even lower, we set the mortality rate of the whole population as the sum of the mortality of the whole population and the mortality of HFMD.
2. Transmission of HFMD occurred via person–person, and the transmissibility between infectious individual and asymptomatic one may be different. So, the *k* was defined as the relative transmissibility rate of asymptomatic to symptomatic individuals. At the same time, we assumed the *S* would be potentially infectious as long as they are in contact with infectious individuals or asymptomatic individuals, and the coefficient of the infection rate was set as *β*.
3. Infectious individuals (I) and asymptomatic individuals (*A*) came from the susceptible individuals, so we considered that there was a certain proportion of exposed individuals *pE* (0⩽*p*⩽1) transformed into *I* after incubation, another part of exposed individuals (1– *p*) *E* were transformed into *A* after incubation as well. At a certain time (t), we set the speed of the development speed from the *E* to *I* pathway as *ω* (0⩽*ω*⩽1), and the development speed of *E* to *A* pathway as *ω*′. So the proportional coefficient of *E* to *I* was set as *pω*, and *E* to *A* was set as (1– *p*) *ω*′.
4. In our model,* I* and *A* may move to *R*, and the speed of recovering was in direct proportion to the number of individuals. The proportional coefficients were *γ* and *γ′* respectively.
5. When *I* and *A* moved to *R*, we assumed that the infectious individuals recovered from the virus type they were diagnosed with, and that these recovered individuals had permanent immunity against this virus: thus, *R* was set as the end of the model.
  $$ \frac{{{{dS}}}}{{{{dt}}}} = {{nbr}} - {{\upbeta { S}}}\left( {{{I}} + {{kA}}} \right) - {{drS}} $$
  $$ \frac{{{{dE}}}}{{{{dt}}}} = {\upbeta { S}}\left( {{{I}} + {{kA}}} \right) - {\omega E} - {{drE}} $$
  $$ \frac{{{{dI}}}}{{{{dt}}}} = {{p\omega E}} - {{drI}} - {\gamma I} - {{fI}} $$
  $$ \frac{{{{dA}}}}{{{{dt}}}} = \left( {1 - {{p}}} \right){\omega E} - {\upgamma }^{{\prime}} {{A}} - {{drA}} $$
  $$ \frac{{{{dR}}}}{{{{dt}}}} = {\gamma I} + {\upgamma }^{^{\prime}} {{A}} - {{drR}} $$
  $$ {{n}} = {{S}} + {{E}} + {{R}} + {{I}} + {{A}} $$

### Parameter estimation

The parameters *β*, *ω*, *ω*′, *γ*, *γ*′, *k*, *p* and *f* represented the infection rate coefficient, incubation period coefficient, latent period coefficient, removal rate coefficient of dominant infection, removal rate coefficient of recessive infection, infectivity coefficient of recessive infection compared with dominant infection, the proportion of dominant infection and fatality rate of dominant infection respectively.

1. The birth rate (*br*) and death rate (*dr*) were collected from 97 counties’ statistical yearbooks in Jiangsu Province.
2. Studies showed that the proportion of dominant infection ranges were 19–47%^2,15,16^, selecting the median value 44.23%, therefore *p* = 0.4423.
3. The ranges of the incubation period (1/*ω*) were 3–7 days^2,17,18^, selecting the median value 5 days, therefore *ω* = 0.2. The latent period was set to 5 days, therefore *ωʹ* = 0.2.
4. The duration of symptomatic infection was 2 weeks^8,18^, therefore, the rate of disease removal *γ* = 0.0714. The duration of asymptomatic infection ranged from 2 to 4 weeks^15,16^, Median of 3 weeks was chosen as the disease removal rate of asymptomatic patients, therefore, *γ*’ = 0.0476.
5. The mortality of symptomatic infection ranged from 0.0001 to 0.0005^19,20^, selecting the median value 0.0003. Parameter *β* is estimated by curve fitting.
6. There is no clear data or references to support the parameter *κ*, which is still uncertain. Therefore, in this study, we assumed *κ* = 1 for calculation, and sensitivity analysis was carried out to calculate its impact on the model.

The significance of each variable and parameter of the model is shown in Table 3.

**Table 3:** Parameter definitions and values of SEIAR model.

Parameter	Description	Unit	Value range	Value	Method
*br*	Birth rate	1	0–1	–	Actual data
*dr*	Death rate	1	0–1	–	Actual data
*β*	Transmission relative rate	individual^-1^·Day^-1^	0–1	–	Curve
*κ*	Transmissibility coefficient of A relative to I	1	0–1	1	–
*p*	Proportion of asymptomatic	1	0–1	0.4423	Actual data
*ω*	Incubation relative rate	Day-1	0–1	0.2	2, 24, 25
*ω′′*	Latent period relative rate	Day-1	0–1	0.2	2, 24, 25
*γ*	Recovery rate of the infectious	Day-1	0–1	0.07143	22, 23
*γ'*	Recovery rate of the asymptomatic	Day-1	0–1	0.04762	22, 23
*f*	Fatality rate of HFMD cases	1	0–1	0.0003	Actual data

### Transmissibility evaluation index

In this study, the population was not completely susceptible and artificially adopted some prevention and control measures, so we chose the effective reproduction number (*R*_*eff*_) to calculate transmissibility. The calculation formula was as follows:

$$ {{R}}_{{{{eff}}}} = \beta {{S}}\left( {\frac{{1 - {{p}}}}{\gamma } + \frac{{\kappa {{p}}}}{{ \gamma^{{\prime}} }}} \right) $$

### Simulation methods and statistical analysis

Berkeley Madonna 8.3.18 software (developed by Robert Macey and George Oster of the University of California at Berkeley. Copyright©1993–2001 Robert I. Macey & George F. Oster) was used for the curve fitting. The fourth-order Runge–Kutta method, with a tolerance set at 0.001, was used to perform the curve fitting.

The coefficient of determination (*R*^2^) was used to assess the goodness of fit. SPSS software (version 13.0; IBM Corp, Armonk, NY, USA) was used to calculate the *R*^2^. We divided the 97 counties into three typical situations using a fast cluster analysis. Linear regression was used to analyze the correlation between the fitting value and the actual reported value. We analyzed the difference in *R*_*eff*_ in different years, regions, and counties using the Kruskal–Wallis H test. We compared the transmissibility of the 97 counties using the rank-sum ratio (*RSR*). The *RSR* process was as follow: The *R*_*eff*_ values from 2015 to 2020 were divided into the mean value in the first half of the year and the mean value in the second half of the year. For the rank principle, the smaller the *R*_*eff*_ was, the larger was the rank. The rank-sum *RSR* was calculated by ranking. Probit was calculated through the *RSR* distribution, and the regression equation of the *RSR* and probit was constructed. The comprehensive comparison results of *R*_*eff*_ in various regions were determined using the regression equation.

## Supplementary Information

Supplementary Information 1.

Supplementary Information 2.

## References

[1] Repass GL, Palmer WC, Stancampiano FF (2014). Hand, foot, and mouth disease: identifying and managing an acute viral syndrome. Clevel. Clin. J. Med..

[2] Koh WM (2016). The epidemiology of hand, foot and mouth disease in asia: a systematic review and analysis. Pediatr. Infect. Dis. J..

[3] Ramdass P, Mullick S, Farber HF (2015). Viral skin diseases. Prim. Care.

[4] Bian L (2015). Coxsackievirus A6: a new emerging pathogen causing hand, foot and mouth disease outbreaks worldwide. Expert Rev. Anti Infect. Ther..

[5] Wang XF, Lu J, Liu XX, Dai T (2018). Epidemiological features of hand, foot and mouth disease outbreaks among Chinese preschool children: a meta-analysis. Iran. J. Public Health.

[6] Huang Z (2019). Seasonality of the transmissibility of hand, foot and mouth disease: a modelling study in Xiamen City China. Epidemiol. Infect..

[7] Chen S (2019). Estimating the transmissibility of hand, foot, and mouth disease by a dynamic model. Public Health.

[8] Takahashi S (2016). Hand, foot, and mouth disease in china: modeling epidemic dynamics of enterovirus serotypes and implications for vaccination. PLoS Med..

[9] Liao Y (2019). Relative transmissibility of hand, foot and mouth disease from male to female individuals. Epidemiol. Infect..

[10] Ma E (2011). Estimation of the basic reproduction number of enterovirus 71 and coxsackievirus A16 in hand, foot, and mouth disease outbreaks. Pediatr. Infect. Dis. J..

[11] Liu W (2019). Forecasting incidence of hand, foot and mouth disease using BP neural networks in Jiangsu province China. BMC Infect. Dis..

[12] Ji H (2012). Epidemiology and etiology of hand-foot-and-mouth disease seen in Jiangsu province from 2008 to 2010. Zhonghua er ke za zhi = Chin. J. Pediatr..

[13] Sheng, Y. Research on the influencing factors and Countermeasures of the imbalance of economic development in southern and Northern Jiangsu %J Technology and Economic Guide. **28**, 241–242 (2020).

[14] Li XW (2018). Chinese guidelines for the diagnosis and treatment of hand, foot and mouth disease (2018 edition). World J. Pediatr. WJP.

[15] Xing W (2014). Hand, foot, and mouth disease in China, 2008–12: an epidemiological study. Lancet. Infect. Dis.

[16] Chang LY (2002). Risk factors of enterovirus 71 infection and associated hand, foot, and mouth disease/herpangina in children during an epidemic in Taiwan. Pediatrics.

[17] Chang LY (2004). Transmission and clinical features of enterovirus 71 infections in household contacts in Taiwan. JAMA.

[18] Du Z, Zhang W, Zhang D, Yu S, Hao Y (2017). Estimating the basic reproduction rate of HFMD using the time series SIR model in Guangdong, China. PLoS ONE.

[19] Yang Z, Zhang Q, Cowling BJ, Lau EHY (2017). Estimating the incubation period of hand, foot and mouth disease for children in different age groups. Sci. Rep..

[20] Zhang J, Kang Y, Yang Y, Qiu P (2015). Statistical monitoring of the hand, foot and mouth disease in China. Biometrics.

[21] Liu W (2015). Spatiotemporal dynamics of hand-foot-mouth disease and its relationship with meteorological factors in Jiangsu Province, China.. PLoS ONE.

[22] Cheng Q (2018). Ambient temperature, humidity and hand, foot, and mouth disease: a systematic review and meta-analysis. Sci. Total Environ..

[23] Luo K (2020). Interaction analysis on transmissibility of main pathogens of hand, foot, and mouth disease: A modeling study (a STROBE-compliant article). Medicine.

[24] Mao LX (2010). Epidemiology of hand, foot, and mouth disease and genotype characterization of Enterovirus 71 in Jiangsu, China. J. Clin. Virol. Off. Publ. Pan Am. Soc. Clin. Virol..

[25] Sheng, S., Kexing, Z. & Binyun, H. Control analysis of the epidemiological and pathogenic characteristics of hand, foot, and mouth disease before and after EV71 vaccination in Xinwu District of Wuxi City%J public health and preventive medicine. **32**, 80–83 (2021).

[26] Yuling, G. Jiangsu Province health level division study%J Rural health undertakings management in China. **38**, 1254–1257 (2018).

[27] Xiang, Z., Lvlin, Z. & Hao, C. Effects of health equalization on resident health and economic growth—Empirical study based on Jiangsu panel data%J East China Economic Management. **28**, 14–18 (2014).

[28] Xiang, Z., Lvlin, Z. & Xi, C. Empirical study on rural public health development and economic growth: Take Jiangsu Province as an example,%J China's health economy. **34**, 51–53 (2015).

[29] Zhang C (2015). Phylogenetic analysis of the major causative agents of hand, foot and mouth disease in Suzhou City, Jiangsu province, China, in 2012–2013. Emerg. Microb. Infect..

[30] Xiaowen, Z. & Guanghui, D. in 2017 Academic Conference on Environment and Public Health and Branch of Environmental Medicine and Health of Chinese Society of Environmental Sciences, The Annual Meeting of the Biochemical and Molecular Toxicology Professional Committee of the Chinese Toxicology Association in 2017. 2.

[31] Jun, H., Jun, Y. & Jinping, C. Progress on new pollutants in Yangtze River estuary and near sea water environment %J Environmental chemistry **33**, 1484–1494 (2014).

[32] Jing, A. *et al.* Investigation of hand, foot, and mouth disease / herpestic pharyngitis in Jiangsu Province. %J Modern Prevent. Med. **47**, 1758–1761 (2020).

[33] An Z (2016). Technical guidelines for the use of inactivated enterovirus 71 vaccine. Chin. J. Vaccines Immun..

[34] Zhu F (2014). Efficacy, safety, and immunogenicity of an enterovirus 71 vaccine in China. N. Engl. J. Med..

[35] Liang Z, Jian L, Chunhan J (2019). Evaluation and projection of climate change in Jiangsu Province based on the CMIP5 multi-model ensemble mean datasets. J. Meteorol. Sci..

[36] Yi L, Miao M (2019). Projection of precipitation over Jiangsu Province based on global and regional climate models. Trans. Atmos. Sci..

[37] Kimmis BD, Downing C, Tyring S (2018). Hand-foot-and-mouth disease caused by coxsackievirus A6 on the rise. Cutis.

[38] Bian L (2019). Hand, foot, and mouth disease associated with coxsackievirus A10: more serious than it seems. Expert Rev. Anti Infect. Ther..

[39] Lu QB (2012). Circulation of Coxsackievirus A10 and A6 in hand-foot-mouth disease in China, 2009–2011. PLoS ONE.

[40] Huang Y (2015). Characterization of severe hand, foot, and mouth disease in Shenzhen, China, 2009–2013. J. Med. Virol..

[41] Blomqvist S (2010). Co-circulation of coxsackieviruses A6 and A10 in hand, foot and mouth disease outbreak in Finland. J. Clin. Virol. Off. Publ. Pan Am. Soc. Clin. Virol..

[42] Montes M (2013). Hand, foot, and mouth disease outbreak and coxsackievirus A6, northern Spain, 2011. Emerg. Infect. Dis..

[43] Notes from the field (2012). severe hand, foot, and mouth disease associated with coxsackievirus A6 - Alabama, Connecticut, California, and Nevada, November 2011-February 2012. MMWR Morb. Mortal. Wkly Rep..

[44] Fujimoto T (2012). Hand, foot, and mouth disease caused by coxsackievirus A6, Japan, 2011. Emerg. Infect. Dis..

[45] Lu J (2014). Hand, foot and mouth disease in Guangdong, China, in 2013: new trends in the continuing epidemic. Clin. Microbiol. Infect. Off. Publ. Eur. Soc. Clin. Microbiol. Infect. Dis..

[46] Yu X (2020). Etiological and intensive surveillance of hand-foot-mouth disease in Suzhou of Jiangsu Province from2017 to 2018. J. Med. Pest Control..

[47] Zhao J, Hu X (2019). The complex transmission seasonality of hand, foot, and mouth disease and its driving factors. BMC Infect. Dis..

[48] Siyu, Y. Study on the Short-term Effect of Meteorological Factors and Air Pollutants on Hand, Foot and Mouth Disease in Shenzhen, Huazhong University of Science and Technology, (2020).

[49] Zuqin D (2020). Study on the short-term effect of air pollution on HFMD transmission takes Wenzhou as an example. J. Xinyang Norm. Univ. Nat. Sci. Ed..

[50] Nan, H. Study on the Spatial and Temporal Distribution of Hand, Foot, and Mouth Disease and the Environmental Exposure Lag Effect in Guangxi Province, He'nan University, (2019).

[51] Xiangxue, Z. The Spatial and Temporal Pattern and Potential Influencing Factors of Hand, Foot, and Mouth Disease in Henan Province, Chang'an University (2019).

[52] Chuwei L, Bowen C, Yuxia M (2021). Analysis of spatial and temporal characteristics of persistent air pollution and Circulation of a typical Event in Jiangsu Province. J. Lanzhou Univ. (Nat. Sci. Ed.).

[53] Hong-jun Z (2015). Determination of mother preached antibody levels of EV71 and CVA16 in the newborn and evaluation of the influence on the HFMD susceptibility in Yancheng. Modern Prevent. Med..

[54] Jin-xiu X, Hui S (2017). Analysis on epidemic situation of hand, foot, and mouth disease and Discussion on prevention and control measures in Baipu town of Rugao City from 2008 to 2016. World Latest Med. Inf..

