# Supplementary figures and images for: Transmissibility of hand, foot, and mouth disease in 97 counties of China
Source: Sci Rep. 2022 Mar 8;12:4103. doi: 10.1038/s41598-022-07982-y (PMC8902910; doi:10.1038/s41598-022-07982-y)

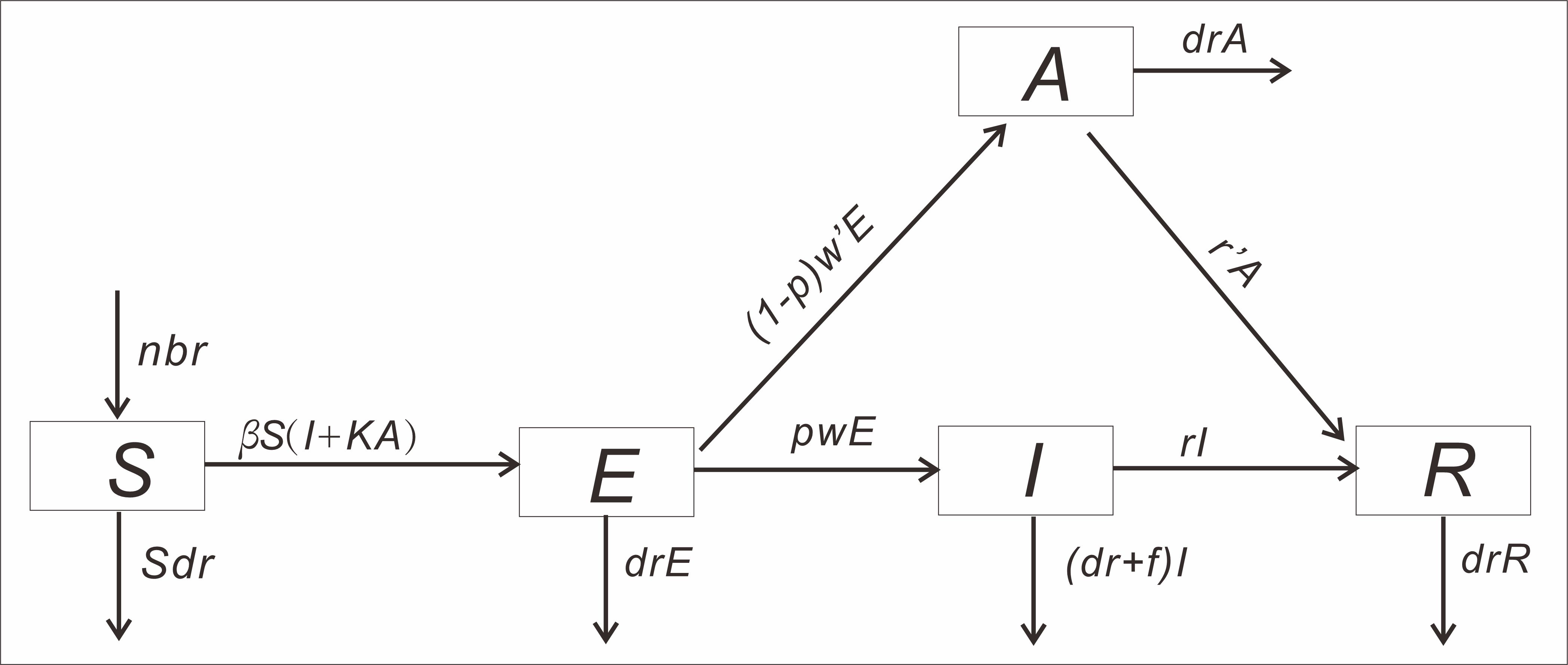

Supplement: Supplementary file 1 — Supplementary Information 1. [file 41598_2022_7982_MOESM1_ESM.jpg]
