# Supplementary material for: Transmissibility of hand, foot, and mouth disease in 97 counties of China
Source: Sci Rep. 2022 Mar 8;12:4103. doi: 10.1038/s41598-022-07982-y (PMC8902910; doi:10.1038/s41598-022-07982-y)
Supplement: Supplementary file 2 — Supplementary Information 2. [file 41598_2022_7982_MOESM2_ESM.docx]

**Manuscript title:** **Transmissibility of hand, foot, and mouth disease in 97 counties of China.**

**Authors: Wei Zhang^1#^, Jia Rui^1#^, Xiaoqing Cheng^2#^, Bin Deng^1^, Hesong Zhang^1^, Lijing Huang^1^, Lexin Zhang^1^,** **Simiao Zuo^1^, Junru Li^1^, XingCheng Huang^1^, Yanhua Su^1^, Benhua Zhao^1^, Yan Niu^3^*, Hongwei Li^1^*, Jian-li Hu^2^*, Tianmu Chen^1^***

**Figure legend**

**Figure S1 Flow chart of the SEIAR model for HFMD**

**Table**

**Table S1 Fitting results of 97 regions in Jiangsu Province**

| Region | City | County | *R^2^* | *P* |
| --- | --- | --- | --- | --- |
| Southern Jiangsu | Changzhou | Jintang | 0.478 | 0.000 |
|  |  | Suyang | 0.416 | 0.000 |
|  |  | Tianning | 0.240 | 0.000 |
|  |  | Wujin | 0.605 | 0.000 |
|  |  | Xinbei | 0.337 | 0.000 |
|  |  | Zhonglou | 0.223 | 0.000 |
|  | Nanjing | Gaochun | 0.733 | 0.000 |
|  |  | Gulou | 0.611 | 0.000 |
|  |  | Jianye | 0.502 | 0.000 |
|  |  | Jiangning | 0.651 | 0.000 |
|  |  | Liuhe | 0.581 | 0.000 |
|  |  | Pukou | 0.579 | 0.000 |
|  |  | Qixia | 0.590 | 0.000 |
|  |  | Qinhuai | 0.521 | 0.000 |
|  |  | Sushui | 0.537 | 0.000 |
|  |  | Xuanwu | 0.499 | 0.000 |
|  |  | Yuhuatai | 0.538 | 0.000 |
|  | Suzhou | Changshu | 0.652 | 0.000 |
|  |  | Gongyeyuan | 0.627 | 0.000 |
|  |  | Gusu | 0.492 | 0.000 |
|  |  | Huqiu | 0.435 | 0.000 |
|  |  | Kunshan | 0.516 | 0.000 |
|  |  | Taichng | 0.495 | 0.000 |
|  |  | Wujiang | 0.532 | 0.000 |
|  |  | Wuzhong | 0.541 | 0.000 |
|  |  | Xiangcheng | 0.498 | 0.000 |
|  |  | Zhangjiagang | 0.543 | 0.000 |
|  | Wuxi | Binhu | 0.616 | 0.000 |
|  |  | Huishan | 0.616 | 0.000 |
|  |  | Jiangyin | 0.816 | 0.000 |
|  |  | Liangxi | 0.595 | 0.000 |
|  |  | Xishan | 0.567 | 0.000 |
|  |  | Xin | 0.621 | 0.000 |
|  |  | Yixing | 0.480 | 0.000 |
|  | Zhenjiang | Dantu | 0.441 | 0.000 |
|  |  | Danyang | 0.581 | 0.000 |
|  |  | Jingkou | 0.511 | 0.000 |
|  |  | Jurong | 0.502 | 0.000 |
|  |  | Runzhou | 0.414 | 0.000 |
|  |  | Yangzhong | 0.519 | 0.000 |
| Northern  Jiangsu | Huaian | Huaian | 0.577 | 0.000 |
|  |  | Huaiyin | 0.575 | 0.000 |
|  |  | Jinhu | 0.508 | 0.000 |
|  |  | Kafaqu | 0.515 | 0.000 |
|  |  | Lianshui | 0.406 | 0.000 |
|  |  | Qingjiangpu | 0.645 | 0.000 |
|  |  | Xuyi | 0.568 | 0.000 |
|  | Lianyun | Donghai | 0.436 | 0.000 |
|  |  | Ganyu | 0.422 | 0.000 |
|  |  | Guannan | 0.590 | 0.000 |
|  |  | Guanyun | 0.379 | 0.000 |
|  |  | Haizhou | 0.505 | 0.000 |
|  |  | Lianyungang | 0.573 | 0.000 |
|  | Suqian | Muyang | 0.528 | 0.000 |
|  |  | Siyang | 0.348 | 0.000 |
|  |  | Sihong | 0.582 | 0.000 |
|  |  | Sucheng | 0.729 | 0.000 |
|  |  | Suyu | 0.673 | 0.000 |
|  | Yancheng | Binhai | 0.467 | 0.000 |
|  |  | Dafeng | 0.410 | 0.000 |
|  |  | Dongtai | 0.412 | 0.000 |
|  |  | Funing | 0.392 | 0.000 |
|  |  | Jianhu | 0.300 | 0.000 |
|  |  | Sheyang | 0.146 | 0.000 |
|  |  | Tinghu | 0.606 | 0.000 |
|  |  | Xiangshui | 0.229 | 0.000 |
|  |  | Yandu | 0.586 | 0.000 |
|  | Xuzhou | Fengxian | 0.377 | 0.000 |
|  |  | Gulou | 0.187 | 0.000 |
|  |  | Jiawang | 0.094 | 0.000 |
|  |  | Peixian | 0.535 | 0.000 |
|  |  | Pizhou | 0.432 | 0.000 |
|  |  | Quanshan | 0.122 | 0.000 |
|  |  | Suining | 0.150 | 0.000 |
|  |  | Tongshan | 0.227 | 0.000 |
|  |  | Xinyi | 0.296 | 0.000 |
|  |  | Yunlong | 0.198 | 0.000 |
| Central Jiangsu | Nantong | Chongchun | 0.787 | 0.000 |
|  |  | Gangzha | 0.792 | 0.000 |
|  |  | Haian | 0.446 | 0.000 |
|  |  | Haimen | 0.598 | 0.000 |
|  |  | Qidong | 0.720 | 0.000 |
|  |  | Rudong | 0.675 | 0.000 |
|  |  | Rugao | 0.496 | 0.000 |
|  |  | Tongzhou | 0.716 | 0.000 |
|  | Taizhou | Gaogang | 0.567 | 0.000 |
|  |  | Hailing | 0.557 | 0.000 |
|  |  | Jiangyan | 0.646 | 0.000 |
|  |  | Jingjiang | 0.806 | 0.000 |
|  |  | Taixing | 0.786 | 0.000 |
|  |  | Xinghua | 0.443 | 0.000 |
|  | Yangzhou | Baoying | 0.503 | 0.000 |
|  |  | Gaoyou | 0.461 | 0.000 |
|  |  | Guangling | 0.465 | 0.000 |
|  |  | Hanjiang | 0.614 | 0.000 |
|  |  | Jiangdu | 0.446 | 0.000 |
|  |  | Yizheng | 0.640 | 0.000 |
